# Evaluation of high-dose rifampin in patients with new, smear-positive tuberculosis (HIRIF): study protocol for a randomized controlled trial

**DOI:** 10.1186/s12879-016-1790-x

**Published:** 2016-08-27

**Authors:** Meredith Milstein, Leonid Lecca, Charles Peloquin, Denis Mitchison, Kwonjune Seung, Marcello Pagano, David Coleman, Elna Osso, Julia Coit, Dante Elmo Vargas Vasquez, Epifanio Sanchez Garavito, Roger Calderon, Carmen Contreras, Geraint Davies, Carole D. Mitnick

**Affiliations:** 1 Harvard Medical School, Boston, MA 02118 USA; 2 Partners In Health, Boston, MA 02215 USA; 3 Socios En Salud, Sucursal-Peru, Lima, Peru; 4 University of Florida, Gainesville, FL USA; 5 St. Georges University, London, UK; 6 Brigham and Women’s Hospital, Boston, MA USA; 7 Harvard School of Public Health, Boston, MA USA; 8 Hospital Nacional Hipólito Unanue, El Augustino, Lima, Peru; 9 Hospital Nacional Sergio E. Bernales, Comas, Lima, Peru; 10 University of Liverpool, Liverpool, UK

**Keywords:** Tuberculosis, Randomized trial, Pharmacokinetics, Rifampi(ci)n, Treatment shortening

## Abstract

**Background:**

Evidence has existed for decades that higher doses of rifampin may be more effective, but potentially more toxic, than standard doses used in tuberculosis treatment. Whether increased doses of rifampin could safely shorten treatment remains an open question.

**Methods/Design:**

The HIRIF study is a phase II randomized trial comparing rifampin doses of 20 and 15 mg/kg/day to the standard 10 mg/kg/day for the first 2 months of tuberculosis treatment. All participants receive standard doses of companion drugs and a standard continuation-phase treatment (4 months, 2 drugs). They are followed for 6 months post treatment. Study participants are adults with newly diagnosed, previously untreated, smear positive (≥2+) pulmonary tuberculosis. The primary outcome is rifampin area under the plasma concentration-time curve (AUC_0–24_) after at least 14 days of study treatment/minimum inhibitory concentration. 180 randomized participants affords 90 % statistical power to detect a difference of at least 14 mcg/mL*hr between the 20 mg/kg group and the 10 mg/kg group, assuming a loss to follow-up of up to 17 %.

**Discussion:**

Extant evidence suggests the potential for increased doses of rifampin to shorten tuberculosis treatment duration. Early studies that explored this potential using intermittent, higher dosing were derailed by toxicity. Given the continued large, global burden of tuberculosis with nearly 10 million new cases annually, shortened regimens with existing drugs would offer an important advantage to patients and health systems.

**Trial registration:**

This trial was registered with clinicaltrials.gov (registration number: NCT01408914) on 2 August 2011.

## Background

Nearly 10 million new cases of tuberculosis (TB) and 1.5 million deaths due to TB occur worldwide each year. Of those who receive treatment, 86 % experience successful outcomes [1]. Although TB treatment is currently recommended for six months, it has been argued that shortening treatment by as little as two months would accrue substantial benefits to patients and health systems [2, 3].

Consequently, studies of shortened treatment have been implemented. These generally fall into three categories. First are studies that have assessed whether shortened regimens would be adequate in patients with less severe disease (represented by absence of cavitation on radiography) [4]. The second group has investigated regimens containing a new drug, pretomanid with or without bedaquiline [5] (STAND:NCT02342886;NC-005:NCT02193776). The third, and most common type has investigated introduction or modification of doses of existing drug classes (fluoroquinolones and rifamycins) in treatment shortening (ReMoxTB, RIFAQUIN, OFLOTUB, TBTC Studies 27, 28, 29) [6–11].

Rifampin (RIF), one member of the rifamycin class, is unique among the drugs explored for shortening potential. It is the only drug that already has a TB indication from multiple stringent regulatory authorities, is globally used routinely for TB, and is cheap and produced by multiple quality-assured generic manufacturers. Additionally, there is an extensive body of in vitro, animal, and human evidence suggesting that higher-than-standard daily doses of RIF may safely and successfully shorten the 6-month TB treatment [12–16].

For TB, RIF is dosed at 600 mg daily (10 mg/kg/day). This dose was selected in the absence of studies optimizing daily dose and in the presence of perceived resource constraints [17, 18]. Although in vitro and animal data revealed concentration-dependent killing [19–25], translation of these findings to clinical studies was influenced by the perceived high cost of rifampin at its introduction. To minimize costs, *intermittent* (1–3 times/week), higher doses of rifampin for TB were explored in humans. Increased plasma concentrations and more rapid culture conversion were observed [26, 27]. Decroix et al. reported that increasing the rifampin dose from 600 to 900 mg resulted in a near doubling of serum concentrations during the two months when concentrations were monitored [26]. Acocella observed tripling of the maximum serum concentration (C_max_) with a doubling of the dose of rifampin, from 10 to 20 mg/kg/day. This is likely due to the significant saturation of rifampin first-pass metabolism as doses were increased [28]. Thus, significant increases in exposure are to be expected from even modest increases in dose. The presence of toxicity among patients receiving intermittent, higher doses of rifampin, however, ended efforts to explore improved activity in the 1970s [29–32]. High-dose (15–20 mg/kg) daily rifampin has now been used for other indications (leprosy [33], resistant *Streptococcus pneumoniae* [34], staphylococcal infections of orthopedic implants [35], *Legionella jordanis* [36], and cutaneous leishmaniasis [37]) without evidence of dose-dependent toxicity. This is consistent with the results of a recently published maximum dose-tolerability study, which found no serious adverse events occurring at doses of up to 35 mg/kg/day for 1- 2 weeks [38]. These observations suggest that the serious toxicities previously associated with high-dose RIF—particularly hepatotoxicity and flu-like syndrome—may be idiosyncratic rather than dose related, or may be linked to intermittency [12, 14–16].

More recent work has corroborated the findings that rifampin exposure is known to be dose-related and at least dose-proportional [39]. One study revealed a near quadrupling of area under the plasma concentration-time curve (AUC)/minimum inhibitory concentration (MIC) when rifampin dose was increased from 300 to 600 mg [40]. In an observational study of Indonesian patients with pulmonary TB by Ruslami et al., AUC increased by a factor of 1.65 when the rifampin dose was increased from 450 to 600 mg [41]. The observed increase in pharmacokinetic exposure with dose has been linked to improved response using markers of efficacy in humans. In studies of early bactericidal activity, a linear increase in the activity of rifampin has been reproducibly demonstrated up to a dose of 1200 mg [42]. This body of evidence supports the concept that rifampin doses greater than 600 mg may increase treatment response during the first two months, which could, in turn, permit treatment shortening.

To address this question, we embarked on a Phase II trial, entitled “Evaluation of high-dose rifampin in patients with new, smear-positive tuberculosis” or, HIRIF. The study is being conducted under investigational new drug application (# 106635) with the US Food and Drug Administration. The study protocol is presented here.

### Objectives

HIRIF has three primary objectives:

1. To assess the difference in steady state pharmacokinetic exposure of rifampin and 25-desacetyl-rifampin across three, daily, oral doses of rifampin (10, 15 and 20 mg/kg/day). This is done through evaluation of AUC_0–24_/MIC of rifampin at steady state.
2. To assess the difference in sputum culture sterilization during the initial 8 weeks across all three rifampin doses.
3. To compare the incidence of grade 2 or higher adverse events related to the study drug during the 8-week intensive phase of treatment, and up to 4 weeks later.

Secondary objectives refine the understanding of safety and efficacy of higher doses of rifampin and explore potential surrogate endpoints for failure and relapse.

## Methods

### Design

HIRIF is a multi-site, randomized, controlled, triple-blinded clinical trial assessing the pharmacokinetics, efficacy, and safety of higher doses of rifampin. Two experimental arms, rifampin 15 and 20 mg/kg/day, are compared to the control arm, rifampin 10 mg/kg/day. The study is randomizing 180 participants to one of three treatment arms in a 1:1:1 allocation. Randomization is blocked, but not stratified. Unblinded pharmacy staff implement treatment assignment and prepare weight-based prescriptions providing only blinded information to participants and other study staff.

**Fig. 1:**
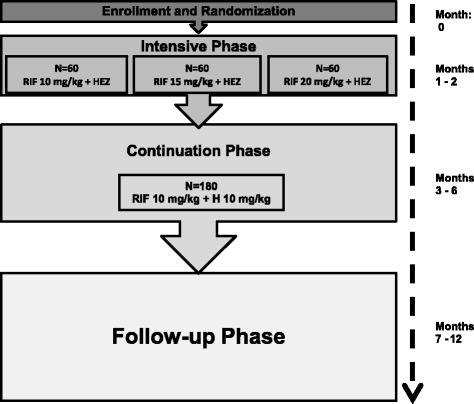
Flow of 12-month study participation

Study participation lasts 12 months. During the first 2 months, participants receive the randomly assigned dose of rifampin 7 days/week, in combination with standard doses of companion anti-TB drugs: isoniazid (H, 5 mg/kg/day), ethambutol (E, 20 mg/kg/day), and pyrazinamide (Z, 25 mg/kg/day). All study participants then receive a 4-month continuation phase of therapy with standard treatment doses (H: 10 mg/kg/day; RIF: 10 mg/kg/day) 3 days/week. Throughout, 50 mg of pyridoxine is administered 3 times/week to prevent peripheral neuropathy, a common side effect of isoniazid. Study participants are followed for 6 months after treatment completion (see Fig. 1).

### Setting

HIRIF is being implemented in two districts in Lima, Peru (Lima Este and Lima Ciudad) where the local implementing partner, Socios En Salud (SES), operates. The year prior to study initiation, approximately 2500 cases of smear-positive TB were reported in the catchment area. Less than 4 % of TB cases were co-infected with human immunodeficiency virus (HIV) and roughly 10 % had diabetes mellitus. Potential participants are identified in 43 peripheral health centers and then referred to either Hospital Nacional Hipólito Unanue (HNHU) or Hospital Nacional Sergio E. Bernales (HNSEB), according to jurisdiction. Both hospitals are research centers certified by the Peruvian National Institute of Health (INS). The National TB Program of Peru endorsed the study and provides oversight and supervision to the recruiting health centers and research centers. Pre-screening sputum microscopy is performed by health center laboratories. Study microbiology is performed by the SES research microbiology laboratory, which is quality assured by the Peruvian INS and the College of American Pathologists. A research pharmacy approved by the Peruvian national regulatory authority and a private clinical lab completed the site resources.

### Study population and eligibility

The study population comprises adults with newly diagnosed, previously untreated, smear positive (≥2+) pulmonary tuberculosis. Patients who meet these criteria on presentation to ambulatory care facilities in the two districts are invited to participate. They are then referred to study staff at the research centers for informed consent and eligibility screening.

Main inclusion criteria for participation in HIRIF are newly diagnosed, pulmonary TB with acid-fast bacilli (≥2+) in a stained sputum smear, and susceptibility to isoniazid and rifampin detected by HAIN MTBDR+ test. Eligible participants are adults (18–60 years old), ≥30 kg, who have never been treated with multidrug anti-TB therapy for more than one month and who have no known intolerance or contraindications to the study drug or companion drugs, and who are not taking any additional drugs for which there may be potential negative drug interactions, synergistic toxicities, or contraindications. Other criteria include a Karnofsky score of ≥50. The following exclusions apply: central nervous system or miliary TB; pericardial or pleural involvement; significant hemoptysis; any uncontrolled condition that may interfere with drug absorption, distribution, metabolism or excretion; uncontrolled diabetes mellitus (glycocylated hemoglobin >7.5 %); serology positive for hepatitis B virus surface antigen or hepatitis C virus antibody; pulmonary silicosis; history of liver disease or current amino alanine transferase greater than 2 times the upper limit of normal (ULN); total bilirubin concentration greater than 2.5 times the ULN, creatinine concentration greater than two times the ULN (or creatinine clearance <60 mL/min), hemoglobin concentration <7.0 g/dL, platelet count <150,000/mm^3^, or white blood cell count <4500 cells/μL. Women of child-bearing potential must not currently be pregnant or breastfeeding and must agree to practice an effective double-barrier method of birth control during treatment. All participants must be willing to undergo HIV testing according to the National Health Guidelines for TB control in Peru; however, patients can be included in the trial regardless of HIV status. Finally, all participants must willingly sign the informed consent form, intend to remain within the jurisdiction of the health center throughout the study to facilitate monitoring and completion of follow-up, and be assessed to be capable of adhering to the study protocol.

### Treatment delivery and retention

All study treatment doses are directly observed. Retention is assured through a system of treatment support and enablers. All treatment is ambulatory and delivered by dedicated directly observed therapy (DOT) supporters. Treatment adherence is assessed through reviews of treatment logs throughout the intensive and continuation phases. Transport costs for study visits are covered by the study. Participants receive regular food vouchers for their participation and meals during prolonged study visits.

### Assessment of study endpoints

The primary pharmacokinetic (PK) endpoint, area under the concentration curve (AUC_0–24_), is assessed through blood sampling on a single day after steady state rifampin exposure is achieved [43] and before the intervention dose is discontinued, between 15 and 56 days post randomization. Participants are randomly assigned to either a sparse (samples are collected pre-dose and at two timepoints after dosing) or intensive (samples are collected pre-dose and at six timepoints after dosing) PK sampling group in a 2:1 allocation. Because AUC_0–24_/MIC is thought to be the pharmacokinetic-pharmacodynamic (PK-PD) parameter best correlated with anti-TB activity [21], the MIC of each participant’s pre-treatment infecting isolate is also estimated from early morning and overnight sputum samples collected at the pre-treatment visit.

The primary efficacy endpoint, change in *M. tuberculosis* log_10_ colony forming units (CFU) in sputum, is assessed by counting log_10_ CFUs in sputum cultures, grown in 7H11 Middlebrook medium. Samples from the same timepoints are also cultured in the BACTEC 960 system (MGIT). These are used to estimate time to culture conversion and change in time to positivity in MGIT. All participants are sampled at baseline, then at 5 time points during intensive phase. Information from sequential pooled sputum samples is used to calculate decline in log_10_ CFUs. Additional early-morning sputum samples are collected for smear microscopy and culture in Löwenstein Jensen medium.

The occurrence of adverse events, use of concomitant medications, and risk of pregnancy and/or breastfeeding are assessed at each study visit. Laboratory screenings are performed to identify hematologic and biochemical abnormalities throughout the study period. Clinical and laboratory findings are graded by clinical investigators according to the modified Adult Toxicity Table [Draft Nov. 2007] for the Division of Microbiology and Infectious Diseases (DMID), National Institute of Allergy and Infectious Diseases (NIAID), National Institutes of Health (NIH).

### Analysis (including power calculation)

The study population of 180 patients (60 per treatment arm) is based on the following assumptions for PK, efficacy, and safety. Existing unpublished data suggest a minimum increase in AUC_0–24_ of 12 mcg/mL*hr across dose groups. Assuming a standard deviation of AUC_0–24_ of 24 mcg/mL*hr and α = 0.05, a linear contrast test across the three treatment groups, the study affords 90 % power to detect a total effect size of 14 mcg/mL*hr between the top and bottom dose levels at a sample size of 50 evaluable subjects per arm, permitting a 17 % loss to follow up.

The estimation of the sample size for the efficacy endpoint is based on computation of the population Fisher’s Information matrix derived from linearization of a non-linear mixed effects model for the data. The parameter, θ_4,_ in this model represents the late phase decay in colony counts, a surrogate measure of sterilizing activity. Under conventional assumptions of α = 0.05 and ß = 0.20 with a coefficient of variation on θ_4_ of 20 %, a sample size of 48 per arm is sufficient to detect a difference between the highest and lowest dose arms of 0.025 log_10_ CFU/ml. This is comparable in magnitude to differences observed in previous colony counting studies. A sample size of 60 per arm allows up to 25 % patient withdrawals.

Among adverse events thought to be associated with rifampin dose, hepatotoxicity is one of the most worrying. Hepatotoxicity has been observed to occur in up to 27 % of patients receiving RIF-containing regimens for TB, with the summary frequency from one meta-analysis estimated at 2.7 % [44]. Other serious toxicities, such as hematologic disorders and flu-like syndrome are estimated to occur in between 1 and 5 % of patients on standard doses of RIF [45]. In a pivotal trial of rifapentine (compared to standard doses of RIF) 5 % of subjects receiving rifampin permanently discontinued treatment [45]. We expect at least 10 % of patients in the control arm to experience a grade 2 or higher event related to RIF. We have 62 % power (1-sided α = 0.1) to detect adverse events occurring twice as frequently in the intervention arms combined, and greater than 95 % power to detect a relative risk of ≥3. Since we are comparing incidence of adverse events, a continuous variable, the statistical power afforded by the study sample size is actually slightly higher.

### Analysis of primary endpoints

The primary PK analysis will be a two-sided linear contrast test of dose-response of AUC_0–24_/MIC across the three dose groups with a significance level of 5 %. Secondary analyses of the summary parameters will include similar tests of dose-response for AUC_o-∞_ and C_max_. Exploratory analyses of additional determinants of the exposure parameters (AUC_0–24_, AUC_o-∞_ and C_max_) and clearance will include as covariates study site, body mass index, sex, and plasma concentration of companion drugs.

Population PK modeling of the parent compound and its major metabolite, 25-desacetylrifampicin, using the rich and complete datasets will be carried out.

The primary efficacy analyses will be two-sided linear contrast tests of the three dose levels on: 1) the parameter θ_4_ (late-phase sterilizing slope) derived from non-linear mixed effects modeling and 2) the hazard ratio of culture conversion in MGIT derived from the Cox proportional hazards model at a significance level of 5 %. These two endpoints, and their performance characteristics for predicting failure/relapse, will be compared with each other, and with the binary 2-month LJ culture conversion endpoint.

To evaluate the effect of dose size on adverse events (AEs) grade 2 or higher, the primary analysis will be a comparison, between the intervention groups and the control group, of time to adverse events occurring in the first 12 weeks after randomization and determined to be related to rifampin. In secondary analyses, the rate of discontinuation due to hepatotoxicity will be compared between the intervention groups and the control group. If a significant difference is detected, comparison will be made between the 15 mg/kg intervention group and the control as well as between the 20 mg/kg intervention group and the control.

### Dissemination of trial findings

The study principal investigators hold primary responsibility for the preparation of publications. Once the trial is complete, the investigators anticipate publishing results of this study in several manuscripts in peer-reviewed scientific journals. In compliance with the policy of International Committee of Medical Journal Editors, this trial is registered in ClinicalTrials.gov: NCT01408914.

## Discussion

The HIRIF trial is a Phase IIB design that incorporates several approaches that are novel in the tuberculosis field. Most importantly, the study is designed to fully support a rigorous pharmacokinetic-pharmacodynamic analysis, which is appropriate to the goals of an early- phase dose-ranging trial. The pharmacokinetic aspect of the study is based on a population approach which is facilitated by the intensive-sparse sampling design and enables pharmacokinetic exposure (AUC_0–24_) to be estimated for all participants in the study. In addition, RIF MIC will be obtained for each participant enabling more direct comparisons with existing preclinical data for the first time in a human clinical trial and accounting for this important source of variability in treatment response [46]. Finally, for increased statistical power [21], the pharmacodynamic outcomes are powered on the basis of quantitative bacteriology, specifically serial sputum colony counting, rather than on the more traditional two-month culture conversion endpoint. A balanced-blocks design of staggered sputum sampling times based on prior studies was adopted to make this approach convenient for patients and logistically feasible for the laboratory [47]. Several alternative biomarkers of treatment response are also being evaluated using these samples.

HIRIF addresses an important evidence gap in the treatment of TB. Although the work that led to the implementation of the 6-month, 4-drug regimen containing daily, 600 mg doses of rifampin was highlighted as a model for medical interventions [48], the optimal dosing for rifampin was overlooked in the series of trials that led to these laudatory remarks. Cost trumped efficacy in the selection of regimens tested in the Medical Research Council trials that led to the development of the current standard of care. That tens of millions of patients may have been subjected to suboptimal doses is an inexcusable tragedy, which must not be repeated in future drug-development efforts. In the case of rifampin, cost is no longer an issue as it is currently produced by several quality-assured manufacturers and sold for pennies per dose: 3.7–4.7 cents per 150-mg rifampicin tablet/capsule, or 7.3–8.5 cents per 300-mg rifampicin tablet/capsule [49].

Results from HIRIF will complement those from other recently conducted studies. These include another Phase II study conducted by the PANACEA Consortium in Tanzania, known as HIGHRIF2 (NCT00760149). This study examined the same doses of rifampin in 180 participants with similar endpoints. Both studies were powered for the PK endpoints, which required smaller sample sizes than efficacy and safety endpoints. Data from the two studies will be pooled to provide greater power to assess dose-related efficacy and toxicity endpoints. PANACEA also has conducted a maximum-dose tolerability study, HIGHRIF1 [38]. Among participants who received up to 35 mg/kg of RIF, both monotherapy and multidrug therapy, there were no serious adverse events. In light of this information, the consortium embarked on a Phase II/III adaptive study, MAMS-TB-01 trial (NCT01785186), that examined rifampin doses of 35 mg/kg in combination therapy. Lastly, RIFATOX (ISRCTN55670677), a toxicity study of higher doses (600 mg vs. 900 and 1200 mg) of rifampin found no association between rifampin dose and toxicity. When HIRIF results are available, we will examine the whole body of recently generated evidence to determine if and what further investigation is necessary. Options include additional Phase II work with higher doses for longer duration than in HIGHRIF1. Also possible is moving forward with Phase III studies of either the 15 or 20 mg/kg dose, depending on results from HIRIF and results pooled with HIGHRIF2. Any proposed study would complement those already conducted or underway (i.e., PANACEA, the proposed RIFASHORT [NCT02581527]). And, it is possible that based on combined efficacy and safety data, there will be no justification for further investigation of higher doses of rifampin for new, smear-positive pulmonary TB. Irrespective of the final outcome, however, we can be confident that an historical wrong has been righted: the dose of rifampin used for TB in the future will reflect the complete evidence on PK, efficacy, and safety assessed using modern methods, available in the 21^st^ century.

## Abbreviations

AE: Adverse events
AUC: Area under the plasma concentration-time curve
CFU: Colony forming units
C_max_: Maximum serum concentration
DMID: Division of Microbiology and Infectious Diseases, National Institute of Allergy and Infectious Diseases, National Institutes of Health
DOT: Directly observed therapy
E: Ethambutol
H: Isoniazid
HAIN: Line probe assay to test for resistance to isoniazid and rifampin
HIRIF: Study acronym
HIV: Human immunodeficiency virus
HNHU: Hospital Nacional Hipólito Unanue
HNSEB: Hospital Nacional Sergio E. Bernales
INS: Peruvian National Institute of Health
IRB: Institutional Review Board
MIC: Minimum inhibitory concentration
N: Number (refers to number of subjects)
PD: Pharmacodynamics
PK: Pharmacokinetics
RIF: Rifampin
SES: Socios En Salud
TB: Tuberculosis
ULN: Upper limit of normal
Z: Pyrazinamide
